# The Role and Value of Chaplains in the Ambulance Service: Paramedic Perspectives

**DOI:** 10.1007/s10943-021-01446-9

**Published:** 2021-10-25

**Authors:** Katie Tunks Leach, Paul Simpson, Joanne Lewis, Tracy Levett-Jones

**Affiliations:** 1 Faculty of Health, University of Technology Sydney, Sydney, Australia; 2 New South Wales Ambulance, Sydney, Australia; 3 School of Health Sciences, Western Sydney University, Penrith, Australia; 4 Faculty of Nursing, Midwifery and Public Health, University of Canberra, Canberra, Australia

**Keywords:** Paramedic, Emergency medical services, Well-being, Chaplaincy, Spiritual care

## Abstract

Chaplains are employed by ambulance services in many states across Australia as one element in a suite of initiatives to support the health and wellness of paramedics. The aim of this paper is to present key findings from a study that explored paramedic perspectives on the role and value of chaplains in the ambulance service. Seventeen paramedics participated in semi-structured interviews. Data were analysed using framework analysis. Two themes were identified: scope of the chaplain’s role and organisational factors influencing the chaplain’s role. Paramedics highly valued what they believed to be proactive and reactive support provided by ambulance chaplains, regardless of paramedics’ personal spiritual or religious beliefs.

## Introduction

Chaplains have long been established in ambulance services around the globe as one aspect of staff health and wellbeing programs. Modern-day chaplaincy teams are made up of multi- or interfaith spiritual care practitioners experienced in supporting staff, regardless of personal beliefs and religious affiliation, through assessment, support, counselling, education, and spiritual or religious care (Carey, 2012; Carey & Cohen, 2015; WHO, 2017). Among the issues which chaplains help staff explore, are values, beliefs, purpose, meaning, hope, forgiveness and personal philosophies (Cunningham et al., 2017; Timmins et al., 2018).

Ambulance chaplains work independently and collaboratively in staff support teams that may also include psychologists, fitness support and peer support officers among others (Ambulance Victoria, 2016; NSW Ambulance Service, n.d.; Queensland Ambulance Service, 2018; St John Northern Territory, 2020). These teams have been implemented by the majority of Australian ambulance services in response to emerging evidence indicating less than optimal psychological, physical, emotional, and spiritual health among paramedics caused by the nature of the work and organisational culture, contributing to paramedics experiencing higher rates of depression, anxiety, post-traumatic stress disorder (PTSD) and suicidal ideation than the average population (Beyond Blue Ltd, 2018; Davis et al., 2019; Lawn et al., 2020; Senate Education Employment References Committee, 2019). These holistic support programs aim to provide a positive response for supporting the needs of staff; however, a dearth of research evaluating their effectiveness is evident (McCreary, 2019).

While chaplains would appear to be a valuable addition to paramedic wellness strategies to address social, emotional and spiritual needs, debate continues in Western cultures on their relevance by those who see the role as solely religious (Best et al., 2021). These views highlight common misconceptions about spirituality, religion and the role of chaplains. A consensus definition developed by Puchalski et al., (2014, p. 646) holds that spirituality is:

“…a dynamic and intrinsic aspect of humanity through which persons seek ultimate meaning, purpose, and transcendence, and experience relationship to self, family, others, community, society, nature, and the significant or sacred. Spirituality is expressed through beliefs, values, traditions, and practices.”

This definition separates religion from spirituality, while still accommodating those holding religious views. It also underpins the work of chaplains in delivering person-centred care for all. Additionally, considerable evidence supports the connection between religion, spirituality and positive health outcomes including enhanced levels of wellbeing, optimism and hope, and reduced rates of depression, anxiety and suicide, along with other positive physical, psychological and social outcomes (Koenig, 2012, 2015). Considering these potential health benefits, maintaining chaplains in the ambulance service may in fact promote positive health outcomes for paramedics (Best et al., 2021; Puchalski et al., 2014). Finally, spirituality is important not only for the paramedic’s personal life but also in their professional practice, through the use of skills including empathy, compassion and resilience which add depth to their role and move it beyond merely technical (Lazarsfeld-Jensen & O’Meara, 2018).

To understand more about how paramedics view chaplains, a systematic scoping review conducted by the first researcher (KTL), on staff perceptions of chaplains in first responder and military settings explored chaplain roles and the perceived value of chaplains; however, the paramedic perspective was noticeably absent from the review (Tunks Leach et al., 2020). The aim of this paper is to present key findings from a study that explored paramedic perspectives on the role and value of chaplains in the ambulance service.

## Methods

### Study Design and Setting

This study was carried out in New South Wales Ambulance (NSWA), Australia in 2020. NSWA employs over 5971 staff (NSW Ambulance, 2020a) and has the largest multifaith ambulance chaplaincy program in Australia. This program employs one paid and 55 volunteer chaplains (23 female and 22 male), including 1 Muslim, 1 Jewish and 53 Christian chaplains. It aims to provide 24 h/7-day support to staff, their families and bystanders, regardless of their spiritual or religious views (NSW Ambulance, 2020b).

This study represents the first phase of an exploratory sequential mixed-methods approach. Underpinned by a pragmatic philosophical framework, this approach allows for multiple ‘truths’ or viewpoints in line with the definition of spiritual care underpinning this study. Pragmatism is driven by the research questions and a commitment to using multiple forms of data to find answers (Biesta, 2010; Creswell & Plano Clark, 2018). This phase consisted of semi-structured interviews with paramedics and will be the basis for developing a survey designed to examine the views of a broader cross section of paramedics in phases 2 and 3 (Creswell & Plano Clark, 2018; Schneider et al., 2016).

As the first researcher is a practicing ambulance chaplain in NSWA, bracketing occurred prior to undertaking any research to document thoughts, assumptions and hypotheses, as well as throughout the research process via reflexive journaling and analytical memos (Tufford & Newman, 2010). To maintain a degree of objectivity, the first researcher also discussed the findings at regular meetings of the broader research team.

### Sample and Recruitment

Following ethics approval, participants were recruited via emails distributed by NSWA’s research unit. Purposive sampling and maximal variation approaches were employed to ensure diverse perspectives were obtained, including people of diverse gender identities, ages, professional experience, city or regional experience and personal spiritual beliefs (Creswell & Plano Clark, 2018; Schneider et al., 2016). Follow-up was by email or telephone to ensure participants met inclusion criteria and understood the interviewer’s role as a chaplain/researcher. Interviews were ceased when no new data were emerging (Coffey et al., 2017; Collins, 2010).

### Ethical Considerations

Ethics approval was obtained from South East Sydney Local Health District Human Research Ethics Committee [2019/ETH13593]. This was then ratified by the University of Technology Sydney Human Research Ethics Committee [ETH19-3820]. Participant information sheets were provided to all participants prior to obtaining written informed consent, and staff were informed that their responses would be deidentified to ensure privacy and confidentiality. Furthermore, they were informed that data would be used in peer-reviewed publications and conference presentations.

### Data Collection

Paramedics were interviewed between July and October 2020. All interviews were conducted by the first researcher in a mutually convenient location and lasted between 30 and 60 min. Eight participants had previously met or worked with the interviewer while nine had not, and all were informed of the first researher's reasons for conducting the research. Interview questions were generated and refined following a scoping review and pilot interviews (Appendix 1). They were provided to participants in advance and used to guide the conversation. The semi-structured interviews allowed the researcher to clarify and pursue themes as appropriate. Each interview was audio recorded and transcribed verbatim.

While some face-to-face interviews were undertaken, the majority took place via telephone due to the research being conducted during the early stages of the COVID-19 pandemic, and the associated restrictions in place. At this stage of the pandemic in NSW, there were very few cases of COVID-19 circulating in the community, with the majority contained to hotel quarantine. Consequently, some chaplains remained in their workplaces while others were removed according to localised restrictions. Therefore, the impact of COVID-19 on chaplain support was not assessed as part of these interviews.

### Data Analysis

Data were analysed using framework analysis, a thematic data analysis method originally developed by Richie and Spencer (1994). This ‘problem focussed’ approach generates thematic groupings, allowing analysis to move beyond the descriptive to the explanatory (Gale et al., 2013; Ward et al., 2013). Transcripts were coded and analysed according to the seven steps outlined by Gale et al. (2013). Regular meetings were held between the research team during the data analysis.

## Results

The sample consisted of 17 permanently employed paramedics; nine identifying as male and eight as female with a mean age of 42 years, and duration of service ranging from two years to 43 years (mean 15.1 years) (Table 1). Nine participants were from Metropolitan Sydney and eight from regional New South Wales. All were qualified paramedics and four had additional specialist qualifications. Four had experience in management roles, two in education roles, two in control centres, and two as Peer Support Officers. Three paramedics were classified by the first researcher as religious (believing in a higher power and attend religious gatherings), four as spiritual but not religious (believing that there might be a higher power but do not regularly attend religious gatherings) and ten as not spiritual or religious (atheist/agnostic/do not believe there is anything out there). Steps were taken to ensure confidentiality, including assigning pseudonyms and removing identifying features of quotes.

**Table 1:** Participant demographic characteristics

Paramedic pseudonym	Gender	Age range	Duration of service	Religion
Paramedic 1	Male	30–39	5–10 years	Not spiritual or religious
Paramedic 2	Male	50 +	20 + years	Not spiritual or religious
Paramedic 3	Male	40–49	20 + years	Spiritual not religious
Paramedic 4	Female	50 +	20 + years	Not spiritual or religious
Paramedic 5	Female	50 +	20 + years	Spiritual not religious
Paramedic 6	Male	40–49	5–10 years	Not spiritual or religious
Paramedic 7	Male	20–29	5–10 years	Spiritual not religious
Paramedic 8	Male	30–39	11–20 years	Not spiritual or religious
Paramedic 9	Male	40–49	11–20 years	Not spiritual or religious
Paramedic 10	Female	40–49	5–10 years	Not spiritual or religious
Paramedic 11	Female	50 +	20 + years	Spiritual not religious
Paramedic 12	Female	20–29	< 5 years	Religious
Paramedic 13	Male	50 +	5–10 years	Religious
Paramedic 14	Female	20–29	< 5 years	Not spiritual or religious
Paramedic 15	Female	20–29	5–10 years	Not spiritual or religious
Paramedic 16	Male	50 +	11–20 years	Religious
Paramedic 17	Female	30–39	5–10 years	Not spiritual or religious

**Fig. 1:**
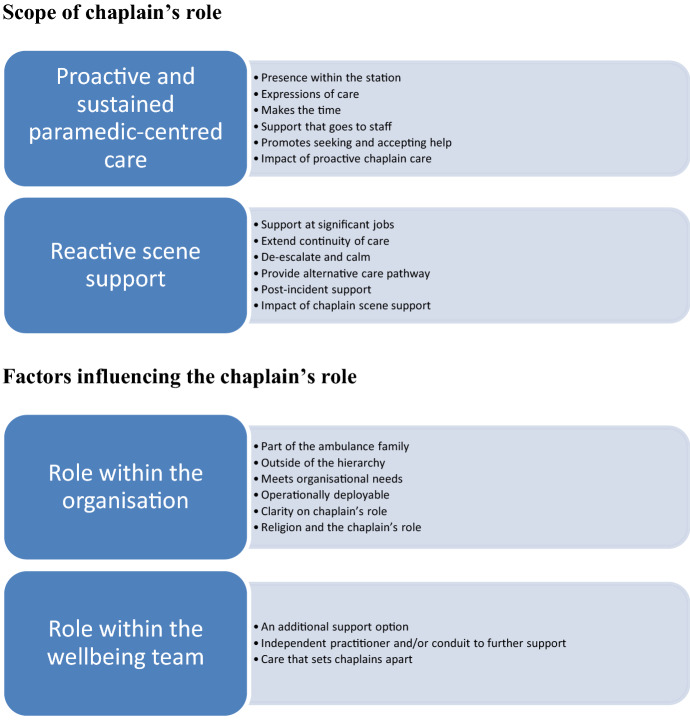
Results Summary

Findings were categorised into two overarching themes: (1) Scope of the chaplain’s role; and (2) Organisational factors impacting the chaplain’s role (Fig. 1).

### Scope of the Chaplain’s Role

#### Proactive and Sustained Paramedic-centred Care

Participants observed that chaplains were proactive, making the time and effort to regularly attend stations and build trust with staff and management. Their activities included hosting barbeques, providing food, or any activity that was perceived to “help create…community” (Paramedic 12). Additionally, paramedics spoke of the value of having someone voluntarily give up their time at any time of the day or night to support them and the powerful message this sends:

“The fact that we've got this group of people who say we value what you do every day enough that we will contribute our own time and resources to just come and help you out, is incredibly valued and impactful for those staff. I think it opens up a heap of conversations that would otherwise not be accessible if someone had just come along and plonked a big program of staff wellness on top of us” (Paramedic 1).

“If everyone had access to one, or just in passing. It doesn't need to be when your house almost burns down in a bushfire. If I'd met [the chaplain] before, I might have been more willing to reach out sooner if I needed something. I might have been able to say to [my manager], look, I don't think I need a psychiatrist, I'm actually having a chat with [the chaplain], over this string of bad jobs, if I'd have known what they were like” (Paramedic 15).

Some participants expressed a desire for more chaplains with greater availability and station presence, however recognised the limitations due to their voluntary role:

“We have to be able to get hold of them. More often than not it goes through the message bank and that is understandable given what they do. They have primary roles and we’re secondary, which is fair enough. Unlike the New South Wales police who use chaplains as a full-time, that’s their job. They are on-call all over the place, they get flown to locations if necessary” (Paramedic 3).

Chaplain support was seen to be mobile, going to paramedics in their workspaces rather than relying on them to make an appointment or attend a specified location. Support included taking staff off-road for coffee, standing with them at significant jobs, meeting them at hospitals, and riding in ambulances with crews to talk in between jobs:

“It would be over a coffee or over a sandwich. We were moving around. It wasn't like they sat us down in the office and then had a talk. Like you might just have a day with [the chaplain] and you'd go and treat on jobs, and you'd do all this and that, but in between is when you talk about that horrific job you did, or I've got some shit going on at home, or whatever. It's in between the jobs, driving to jobs” (Paramedic 15).

These pre-existing relationships and activities undertaken by chaplains were seen to promote conversation and help-seeking, and normalise supportive conversations. Furthermore, participants felt safer divulging personal information to a chaplain they knew and were familiar with:

“When I was in a bit of a rough patch myself. It was just hard to think through the process of who I needed to contact to get some help. When you’re in that sort of frame of mind where everything’s really difficult…reaching out to someone that you don’t know is alienating. It’s terrifying. It’s not something that you’re going to do. Having someone that you’re familiar with and has been on-station that you’re comfortable with, that you can just – you know you’ll call, and something will happen. The help you need will be started” (Paramedic 14).

Some participants reflected on incidents where they perceived that chaplain intervention prevented or minimised staff leaving on workers’ compensation or sick leave, and two instances where chaplain potentially prevented staff from dying by suicide:

“I think personally from having used [a chaplain] in a situation whereby we had serious concerns for the physical and mental wellbeing of one of our staff - I think five years ago that person could have easily killed themself. Easy because they were just so, so over the edge. I don't think you could put a value on that. Absolutely extraordinary. Even if their mental and physical health improved somewhat, they wouldn't have lasted in the job without having that support from the chaplain” (Paramedic 8).

Particular reference was made to the additional value chaplains with clinical experience bring, such as paramedicine or nursing. This experience was perceived to promote faster connections, and a more holistic understanding of the paramedic in their role and life:

“You’ve got the chaplains that are obviously clinically minded as well, so they’re even more in tune with what’s going on. I think that’s really important. I’m not saying the best chaplains are nurses or paramedics, but I do think that’s important because they have a good understanding of what we’re doing medically as well… they can talk about the whole thing, not just about their emotions” (Paramedic 11).

#### Reactive Scene Support

Paramedics also spoke of support provided by chaplains called out to significant jobs or ‘on-scene’. These include major incidents, patients known to paramedics, high profile jobs reported in the media, and jobs eliciting strong emotions or with personal impact (e.g. paediatric cardiac arrests and death by suicide):

“We've got all these staff psychologists and it's another tool to add to the belt for staff welfare, but they’re only available Monday to Friday between those hours…I know that if it's two in the morning and shit goes down, I can call the number and I can get a chaplain in ambulance uniform on the scene within whatever timeframe. I think that is just absolutely invaluable” (Paramedic 8).

Chaplains were also seen to bridge a perceived gap in care between paramedics and bystanders, extending the ambulance continuity of care beyond the clinical. Paramedics spoke of the value in chaplains who de-escalated bystanders or brought a sense of calm, supported people who witnessed the trauma, answered questions, provided grief support, and connected bystanders to external support services. In some instances, chaplains provided an alternative pathway of care for patients who could not be left alone but did not necessarily need hospitalisation. Paramedics said it gave bystanders someone to connect with and enabled them to maintain focus on the patient:

“A young guy came home to find his dad dead. There was nothing for us to do as ambulance paramedics because it was already done, and the chaplain came and sat with this family. They had no-one but themselves and the chaplain there, and it was just beautiful… It's bridging that gap of care. And it's not clinical care, it's compassionate care and love and warmth, and making them feel that they’ve got something and someone to talk to” (Paramedic 11).

Post-incident support was also identified as part of the chaplain’s role. Paramedics spoke of the value in knowing chaplains would check in on them after “calamitous sad stuff” (Paramedic 1) in person and via phone. Support could be formal, such as at post-incident clinical debriefs, or through informal conversation. It was noteworthy that paramedics did not necessarily want to talk about the job at the debriefing:

“We went to the train station where unfortunately the bloke died. We got a coffee afterwards. The chaplain had taken us offline for a short period and it was only 20 minutes or so but I don't think we talked about anything about the job, but I really took comfort in that. That was brilliant. A couple of days later, I might not be feeling as good potentially. It's always nice just to have a [follow up] phone call” (Paramedic 7).

Interviewees frequently attached an element of emotion or impact to their reflections. Words like ‘worry’ or ‘guilt’ were associated with needing to focus on the patient and forgo bystander support or needing to leave bystanders with no support after someone has died. Others reflected on the personal impact of constant exposure to a trauma. Having chaplains attend to take over patient/bystander care or turning up with a tray of coffees was perceived to make a positive impact, with participants using phrases like ‘you can get closure’, ‘you can let go’, and ‘they are our pressure release valve’:

“It's like when we walk out in past years, we just shut a door and we go, sorry we had to meet you under those circumstances. But having someone there to hold their hand at the end of the day. It's like we leave them we say, hey, we're tapping out, you're tapping in. There's a sense of, you're just not walking away and leaving them by themselves. It tugs at your heart, but now you go, actually there's a person I can leave to take that over for me so I can go back out and do my job” (Paramedic 11).

Some paramedics reflected on the value of talking to chaplains in the aftermath of significant trauma (e.g. paediatric death, suicide) and engaging in “no-holds-barred conversation”. They reflected on the value of having someone available to listen and let them get things off their chest ‘without agenda’ or ‘feeling they were being diagnosed’, and how this validated their experiences: “It's about feedback. Our feelings are quite normal in extreme situations, and they're happy to talk to explain, and there's no ridicule” (Paramedic 2). The listening element was especially valued by most participants in these conversations:

“I didn't feel like I needed to see a psychiatrist. Sometimes you just need to talk. Sometimes you don't need anyone to say anything to you about it, but just go, that's terrible. You've been through hell, seeing that. How do you feel? Then you talk about it. I don't need any answers. I don't need help” (Paramedic 15).

Additionally, these conversations were valued because participants felt they could talk about confronting content or topics that otherwise could not be discussed with family or friends outside of work. However, some participants were concerned about the impact of these conversations on chaplains:

“We are offloading onto the chaplains the worst of the worst, worst shit that we go to and see or have to deal with. We're not just giving them the little fluffy shit to deal with. It's the worst. That's what I worry about, that they are going to lose it or go crazy” (Paramedic 8).

### Organisational Factors Influencing the Chaplain’s Role

#### Role Within the Organisation

Embedding chaplains within the organisation was overwhelmingly viewed as positive if they met paramedic needs. Participants spoke of a protective culture suspicious of outsiders, and how having someone ‘on the inside’, in uniform and easily recognisable to paramedics, promoted the idea that chaplains are part of the ambulance family:

“I think we're incredibly resistive to outsiders. Massively. Even other emergency services. We hate everyone, but to have someone turn up in an ambulance uniform with the roundel on their shoulders knowing that you've come in and you've seen the same shit that we have, you've been in the trenches with us, I think there's a respect gained there” (Paramedic 8).

“When we speak to each other, we speak to each other. We speak our own language. In Emergency Services we live and breathe a life that nobody else sees…A chaplain in the organisation is - it's someone else to talk to” (Paramedic 13).

Paramedics expressed the importance of chaplains being outside the hierarchy, reflecting on the value of having support that was not management or clinical, rather someone whose sole focus was paramedic welfare:

“They're completely outside the chain of command, which makes them fully independent. That's why the chaplains coming in, they do not represent management. The people can actually have a bitch about processes to be empathetically listened to without being judged, or ‘toughen up, young lad, that's life’. You can't put a price on that” (Paramedic 2).

Having a workforce that met organisational needs and was operationally deployable promoted the idea that chaplains could support staff and bystanders ‘on the front line’. Paramedics appreciated being able to call for chaplains to be deployed on-scene. They also valued having diverse genders of chaplains, a chaplain team that could mobilise rapidly to meet demand, and the desire for more of them:

“Often on the way to a job if they think it’s complicated or complex enough, some of them will ask, is there any chance we can get a chaplain? Give a chaplain a heads-up now and we’ll let you know if we need them. I've done that before too” (Paramedic 3).

There was a lack of clarity around the chaplain’s role throughout most interviews. Many participants could not explain what chaplains do, and in some instances did not know chaplains existed until they met them on their station or on-scene. Other participants stated the chaplain’s role included emotional support, staff welfare, to meet spiritual needs and educate staff on topics including grief:

“It comes down to paramedic wellbeing is the main function. I also sort of thought that if someone is a person of faith and they witness a horrific scene and they have trouble reconciling that, I think the chaplains can be a bit of a stepping stone. I don’t have faith. I see a bad thing, I think that’s just a bad thing” (Paramedic 10).

For a considerable number of participants, there was a lack of clarity around the role religion plays in the chaplain’s activities. Several paramedics stated that prior to knowing a chaplain they were concerned about having religion pushed on them, about negative public sentiment towards the church, and how the religious symbolism on the chaplain’s uniform was off-putting. After meeting the chaplain, others were confident that there was ‘no denominational component’ to chaplain activities unless requested. Those who identified as religious appreciated opportunities to discuss their job and faith:

“Given that we are dealers in life and death, and professionally we spend our time fighting God over our patients, it’s important to have some sort of spiritual guide there, I think. Someone that’s involved” (Paramedic 16).

#### Role within the Wellbeing Team

Paramedics saw value in having a greater range of choices should they decide to reach out for help, acknowledging that no single service was going to meet everyone’s needs. Despite acknowledging the organisation is promoting psychology as a confidential treatment option, suspicion remained for some due to negative historical experiences:

“I trust the chaplains. I trust the chaplaincy service more than I trust the psychological service. I know they're trying to break down those barriers, and I understand that. I just don't know if I want to risk it. So, without the chaplaincy there, I would be in a pickle. … It's just comforting to know it's there if I need it, and that I can trust him” (Paramedic 5).

Paramedics spoke of chaplains who could function independently, and as a conduit to further care (e.g. psychology, management) if needed:

“[Chaplains] are keen to listen and – but then also willing to take action. Like I will listen to you, but I can put this in place or get you in contact with someone else. Whatever you need, I’ll get started…I think having that understanding of the way we work and then being linked into the other support structures that exist and then the operational structures, so that you can talk to our managers and that sort of thing is absolutely pivotal” (Paramedic 14).

When reflecting on what differentiates chaplains from other support options, paramedics spoke of chaplains “providing a more human level of engagement than [other services] can sometimes provide” (Paramedic 9), a role that is “about paramedic support first and foremost” (Paramedic 7), and knowing that someone is there “that’s got my back” (Paramedic 5). Paramedics reflected on the value of chaplains who ‘get it’ or understood the language and pressures because of shared knowledge and experiences. Consequently, staff felt it made it easier to accept support and the chaplain’s input as valid:

“I’ve actually had a psychologist who said, ‘yes, I know what you mean’. No, you don’t. Or, ‘I know how you feel’. You haven’t got a clue how I feel. This [chaplain], they know exactly how I feel. They can be at a job standing next to you and if they said, ‘I get what you mean’, they actually did get what you meant” (Paramedic 4).

The final question in each interview asked if participants thought chaplains add value to the ambulance service?” Sixteen of the seventeen participants expressed in the affirmative. The remaining participant had previous experiences with chaplains engaged in unprofessional behaviour and believed chaplains did not meet their needs. Their response to this question was “I think it comes down to the chaplain…they didn't meet my need and I wouldn't consider them” (Paramedic 17).

## Discussion

This study explored paramedic perspectives on the role and value of chaplains in the ambulance service. Data analysis identified that paramedics valued what they perceived to be proactive and reactive support provided by ambulance chaplains, regardless of their personal spiritual beliefs. Several findings in this study were new owing to the lack of peer-reviewed research specific to ambulance chaplaincy, while others were supported by research in similar settings such as first responder, military and hospital emergency/critical care environments. Additionally, findings aligned with the World Health Organisation’s (WHO) Spiritual Intervention Codings of religious/pastoral/spiritual assessment, support, counselling/guidance, education and ritual (Carey & Cohen, 2015; WHO, 2017).

Results relating to the role of ambulance chaplains were clustered around two significant themes: relationships and professional capability. Relationships were built through proactive activities undertaken by the chaplain within ambulance stations, at hospitals or more broadly in their assigned areas, and occurred outside of operational work. Chaplains physically and emotionally present within ambulance stations provided social, psychological, emotional and spiritual support, and provided spontaneous opportunities for paramedics to engage in conversation, therapeutic or otherwise. This care was valued because it was not geographically removed from paramedics, rather it was situated in their workplace and humanised staff support for paramedics, consistent with narratives and reports from other first responder services (Calams, 2017, August 22; Cunningham et al., 2017; Myers, 2019, March 6; Phoenix Australia, 2016).

Relational support (as opposed to managerial, operational or clinical) was important to paramedics. They valued having someone available who was not there to diagnose, critique clinical performance or fix, but to listen, offer words of reassurance and to connect them to further support if required, which in turn promoted rapport and trust. Like similar studies, paramedics perceived this to reduce barriers to accessing support, normalise help-seeking conversations and make it easier to disclose personal information (Beyond Blue Ltd, 2018; McCormick & Hildebrand, 2015; Moosbrugger, 2006). Some paramedics suggested that pre-existing relationships meant that chaplains knew staff well enough to recognise when something was amiss and to proactively ask them if they were ok.

Professional capability of chaplains was established through a combination of organisational and individual factors. Recruiting a diverse group of volunteer chaplains who sought to be available around-the-clock, providing them with uniforms and building capacity to dispatch them alongside staff to ‘the front line’ promoted the perception that the chaplain’s role was to support staff from within the organisation and mitigated against paramedic self-described suspicion of outsiders. As an organisation built on rank and hierarchy, situating chaplains outside the chain of command was perceived as important and promoted the idea that chaplains were not management and therefore more trustworthy.

Training chaplains to provide on-scene support to paramedics and bystanders was identified as another element of the capable chaplain. This was seen to ‘bridge a gap’ in the ambulance model of care by taking over bystander support, physically and emotionally freeing paramedics to focus on patient care. This was similar to hospital chaplains who cared for and supported patients, their families and organisational staff (Cunningham et al., 2017; Timmins & Pujol, 2018). Some paramedics noted the ‘relief from guilt’ and ‘worry’ chaplains provided in these instances. Further exploration of how these relate to factors preventing or treating burnout, moral distress and injury, compassion fatigue or PTSD and promote post-traumatic growth is warranted (Carey & Hodgson, 2018; Carey et al., 2018; Hylton Rushton, 2018; Tedeschi et al., 2018). Chaplain involvement in post-incident clinical debriefs and follow-ups was also important for paramedics. Giving staff the opportunity to discuss normal emotions in these abnormal events, or simply giving paramedics the opportunity to ‘grab a coffee’ and engage in informal conversation, even if the incident was not directly discussed, was valued. These activities hold similarities to models of psychological first aid, suggesting this may be another element to the chaplain’s role (Australian Red Cross, 2020). Relationship with chaplains was also important in this space, as paramedics on-scene found it easier to work alongside chaplains they already knew and trusted, and saw as professionally capable.

This relational and capability-building approach to the ambulance chaplain model of care promoted the idea that chaplains genuinely understood the pressures participants faced. Because they supported paramedics in all areas of their practice, including on the frontline or in the ambulance trucks in between jobs, chaplains were seen to genuinely ‘get it’ when some other support options were not. While some of these findings represent new evidence, there are similarities with police and military chaplain research (Davie, 2015; Gouse, 2017).

A noteworthy theme underpinning several interviews was the value in chaplains with clinical experience, such as paramedicine or nursing. These chaplains were valued for their ability to engage with staff beyond the spiritual and emotional to incorporate a more holistic view of the clinician, including understanding of common terminology and humour. Literature from hospital critical care settings examining the role of clinically trained chaplains shows promising correlations with these findings (Cunningham et al., 2017; Timmins & Pujol, 2018). Exploration of a clinical chaplain or ‘expert companion’ model of care may provide benefits in areas such as promoting moral resilience and post-traumatic growth through having chaplains in ambulance vehicles working alongside and supporting staff at, and in the immediate aftermath of, clinical care (Hylton Rushton, 2018; Tedeschi et al., 2018). Additionally, promising results from military literature have shown chaplains act as first-line providers of care and facilitate staff connecting to further mental health support (Besterman-Dahan et al., 2012).

As part of a staff health team, chaplains were valued as an additional support option. Paramedics stated that no single staff support service would meet everyone’s needs, and having chaplains available gave them more choice. Chaplains’ ability to care for paramedics individually and confidentially, yet also act as a conduit to help staff access additional support, was valued. Some paramedic stories explored the chaplain’s role in supporting staff returning from worker’s compensation/leave, or preventing suicide or significant harm, with some staff powerfully moved when recounting the impact of these interventions. For some paramedics, historic stigma associated with accessing other formal support such as psychology remained, and chaplains remained their preferred way to access help, which was consistent with other findings (Phoenix Australia, 2016; Senate Education Employment References Committee, 2019).

While no one staff support role is truly independent of others, findings from this study suggest paramedics see chaplains as offering a unique skillset. The participants indicated that ambulance chaplains established relationships, maintained close proximity to staff, were clinically capable and frequently available around-the-clock. Furthermore, participants saw chaplains as a support option who, through shared experiences, genuinely understood paramedic life. They were trusted to support emotional, spiritual and welfare needs when staff did not feel comfortable discussing these with other support services, yet also able to triage and guide staff towards additional support if required. However, it is important to note these are preliminary findings and further research on the views of both chaplains and a wider paramedic cohort will be valuable to consolidating these findings.

Two recurring themes were identified across interviews that limited or inhibited chaplains in their role. On one hand, the voluntary nature of chaplains in this service ‘sent a powerful message’ to paramedics about how much chaplains valued their welfare, yet many simultaneously made statements wishing there were more chaplains with greater availability. For many paramedics this limited their access to chaplains because they feared disturbing chaplains in their paid roles outside of NSWA, or the chaplain was not available when they wanted them. This was a new finding not seen in military or other first responder data and warrants further investigation as it suggests a negative impact on capacity building of chaplains for the current and future ambulance service.

Another significant inhibitor of staff accessing a chaplain was not understanding the chaplain’s role, and the role religion plays in their activities. Paramedics held concerns about chaplains evangelising, and the wearing of religious symbols on their epaulettes further contributed to these concerns suggesting consideration should be given to the use of religious symbols on uniforms. While relationships appeared to overcome some of these reservations, these visual cues and the lack of clarity from the outset on the chaplain’s professional role and responsibilities within the ambulance service generated reservations. These findings hold similarities to police, military and healthcare chaplaincy literature (Best et al., 2021; Moosbrugger, 2006; Roberts, 2016; Snowden, 2021). To address this, ambulance chaplains should also consider taking steps to establish minimum education requirements, standards of practice and affiliation with professional health and chaplaincy organisations, and organisations should develop and promote clear role descriptions in line with their specialised chaplain workforce (Association of Professional Chaplains, 2021; Best et al., 2021; Spiritual Care Australia, 2020; Timmins et al., 2018).

Despite expectations that participants in this study may primarily be spiritual or religious paramedics, it was noteworthy that ten of the seventeen were not spiritual or religious yet still valued chaplaincy. This suggests that personal spiritual beliefs may not be a barrier to accessing chaplain support. Evidence describing those who utilise chaplains and the personal spiritual beliefs they hold more broadly is scarce, however these findings are similar to findings from military and hospital literature (Hale, 2013; Ormsby et al., 2017). One recent study suggested people positively evaluated chaplain encounters even in the absence of faith concordance, with key factors like relationship building and a move from ‘religious’ motivations to more spiritual or existential approaches contributing to this perspective (Liefbroer, et al., 2021).

## Limitations

While the ambulance service in this study was selected for the large size of its chaplaincy team, confining the interview cohort to a single ambulance service impacts its generalisability. Phase three of this exploratory sequential study will be important to test these findings more widely to determine if they are generalisable to the wider paramedic population. Additionally, the perspectives of chaplains themselves were absent from this paper, and there was a lack of representation from people of faiths beyond the Judeo-Christian population.

In addition to addressing the limitations outlined above, opportunities for further research may include establishing and evaluating education programs for frontline chaplaincy, exploring novel approaches to the provision of chaplaincy care including incorporating clinically trained chaplains, and exploring chaplain collaboration with other mental health professionals in prevention and treatment of psychospiritual and emotional aspects of conditions including moral distress and injury, and PTSD—an area showing promise and favourable results in the military setting (Carey & Hodgson, 2018; Starnino et al., 2019).

## Conclusion

The chaplaincy program examined in this study appeared to be a proactive and reactive support option available to staff in their workspaces. Built on a foundation of relationships, trust and a ‘reaching in’ approach, chaplains provided opportunities for formal and informal conversation, the provision of psychological first aid, and facilitation of access to further support when required—a ‘first responder’ to the first responders. Additionally, by situating trained and equipped chaplains within the ambulance service to respond alongside paramedics enabled chaplains to provide on-scene support and extend the ambulance continuity of care for staff and bystanders. Through their presence they potentially protect staff from strong emotions generated by morally and emotionally challenging encounters, and enhance the paramedic’s capacity to focus on patient care. While work remains to be done educating staff on the chaplain’s role and the evidence underpinning spiritual care, as well as clarifying the place of religion in chaplain activities, and on testing these findings on a larger and more diverse cohort, these results suggest chaplaincy is a valued spiritual, emotional and social support option for staff in ambulance services.

## Data Availability

Requests for access to the framework matrices can be made to KTL after submission of her thesis.

## Appendix 1: Interview Questions

The following questions were used to guide the semi-structured interviews. They may or may not have been asked in the order listed, depending on the flow of the conversation:

1. Can you tell me about your experience or experiences with a chaplain?
2. What do you understand the chaplain’s role to be?
3. What skills and attributes should chaplains possess to be effective?
4. Do you think the chaplain is valuable to the organisation? Why or why not?
5. Do you think the chaplain is valuable to you personally? Why or why not?
6. Do you think your interactions with a chaplain have impacted your wellbeing? If so, how?
7. Is there anything else you would like to tell me more about in relation to chaplains, or anything else we have discussed today?
